# A Novel Small Peptide Inhibitor of NF*κ*B, RH10, Blocks Oxidative Stress-Dependent Phenotypes in Cancer

**DOI:** 10.1155/2018/5801807

**Published:** 2018-11-04

**Authors:** Jessica Gambardella, Michele Ciccarelli, Carmine Del Giudice, Antonella Fiordelisi, Matteo De Rosa, Marina Sala, Roberto Pacelli, Pietro Campiglia, Bruno Trimarco, Guido Iaccarino, Daniela Sorriento

**Affiliations:** ^1^Department of Medicine, Surgery and Dentistry “Scuola Medica Salernitana”, University of Salerno, Via Salvatore Allende, 84081 Baronissi, Salerno, Italy; ^2^Department of Advanced Biomedical Sciences, Federico II University, Via Pansini 5, 80131 Naples, Italy; ^3^Department of Pharmacy, University of Salerno, Via Giovanni Paolo II 132, 84084 Fisciano, Salerno, Italy

## Abstract

**Background:**

The RH domain of GRK5 is an effective modulator of cancer growth through the inhibition of NF*κ*B activity. The aim of this study was to identify the minimum effective sequence of RH that is still able to inhibit tumor growth and could be used as a peptide-based drug for therapy.

**Methods:**

Starting from the RH sequence, small peptides were cloned and tested in KAT-4 cells. The effects on NF*κ*B signaling and its dependent phenotypes were evaluated by Western blot, TUNEL assay, proliferation assay, and angiogenesis *in vitro*. *In vivo* experiments were performed in KAT-4 xenografts in Balb/c nude mice.

**Results:**

A minimum RH ten amino acids long sequence (RH10) was able to interact with I*κ*B, to increase I*κ*B levels, to induce apoptosis, to inhibit KAT4-cell proliferation, NF*κ*B activation, ROS production, and angiogenesis *in vitro*. *In vivo*, the peptide inhibited tumor growth in a dose-dependent manner. We also tested its effects in combination with chemotherapeutic drugs and radiotherapy. RH10 ameliorated the antitumor responses to cisplatin, doxorubicin, and ionizing radiation.

**Conclusion:**

Our data propose RH10 as a potential peptide-based drug to use for cancer treatment both alone or in combination with anticancer therapies.

## 1. Introduction

Radiotherapy and chemotherapy are the currently available therapeutic treatments for cancer [1]. In the last decade, the identification of specific molecular targets that are associated with cancer has posed the basis for alternative therapies [2–4]. To date, several targeted therapies, such as hormone therapy [5], signal transduction inhibitors [6, 7], gene expression modulators [8], apoptosis inducers [9], angiogenesis inhibitors [10], and immunotherapy [11], entered the clinical scenario and improved the outcomes of cancer patients. In the quest for new molecular targets, the transcription factor NF*κ*B is putatively interesting since it is involved in the regulation of several processes (proliferation, apoptosis, differentiation, angiogenesis, and inflammation) that favor cancer development and progression [12, 13]. Indeed, NF*κ*B exerts protumorigenic effects in several human cancers [14–23]. Moreover, it is involved in the development of resistance to therapy since it protects cells from apoptosis induced by tumor necrosis factor alpha, ionizing radiation, and chemotherapeutics [24]. Targeting NF*κ*B activity in cancer is, therefore, a promising anticancer strategy. We have previously identified a new inhibitor of NF*κ*B activity, the RH domain of G protein-coupled receptor kinase 5 (GRK5), that is effective in several cell types, including cancer cells [25–28]. Indeed, the RH domain of GRK5 binds I*κ*B*α*, the main inhibitor of NF*κ*B, blocks its degradation in response to stimuli, and keeps NF*κ*B in an inactive state [25]. The strategy is effective both in cancer [26] and in cardiac cells [27]. However, the full-length sequence of the RH domain includes 120 amino acids, a too large protein to allow the development of small molecules for therapeutic purposes. Thus, the aim of this study is to identify the minimum effective sequence of the RH domain of GRK5 that could become a useful peptide-based drug in the treatment of cancer by means of NF*κ*B inhibition.

## 2. Materials and Methods

### 2.1. Cell Culture

Human tumor cells (KAT-4) were a kind gift of Prof. Illario (University Federico II of Naples, Italy). Bovine aortic endothelial cells (BAEC) were purchased from Sigma-Aldrich. Cells were tested for mycoplasma contamination. Cell lines were cultured in Dulbecco's Minimal Essential Medium (DMEM) supplemented with 10% foetal bovine serum (FBS) at 37°C in 95% air–5% CO_2_.

### 2.2. Plasmid Constructs

In order to synthesize small parts of the RH domain of GRK5, we used pcDNA3.1-GRK5-RH [25] as a template to amplify three overlapping 70 amino acids sequences. HIND-III and XBA-I restriction sites sequences were used for cloning.

RH#1 FOR: 5′TAAGCTTGGATGTGCGAAACCAGAA3′

RH#1 REV: 3′CCCTCTAGATTGGGTGATGAAAAC5′

RH#2 FOR: 5′TAAGCTTGGATGGAAGTTACTCCAG3′

RH#2 REV: 5′CCCTCTAGATTCTTTGCAGGGTTT3′

RH#3 FOR: 5′TAAGCTTGGATGACCCCAAAGTCC3′

RH#3 REV: 5′CCCTCTAGAGTACTCGTGGAAGGGTT3′

Amplified sequences were purified by gel extraction kit (Invitrogen) and cloned into pcDNA3.1-Myc/His vector (Invitrogen) by means of T4 DNA ligase (Promega).

### 2.3. Peptides Synthesis

Based on the cloning of RH fragments, we identified a minimal sequence of 10 aa that possibly retains the RH biological effects. We, therefore, designed a 10 aa peptide based on this sequence conjugated to TAT sequence (RH10) and a control peptide based on a flanking inactive sequence conjugated with TAT (CTRL−). Peptides were synthesized using an automated microwave peptide synthesizer from Biotage AB (Initiator + Alstra) on a Rink-Amide-ChemMatrix resin (0.250 g, loading 0.48 mmol/g). All couplings were achieved for 10 min at 75°C (2×) using a coupling reagent HBTU (3 equiv, 0.6 M), HOAt (3 equiv, 0.5 M), and DIEA (6 equiv, 2 M) in N-methyl-2-pyrrolidone (NMP). The N*α*-Fmoc protecting groups were removed by treating the protected peptide resin with a 25% solution of piperidine in DMF (1 × 3 min, 1 × 10 min) at room temperature. Finally, the peptides were released from the resin with TFA/TIS/H_2_O (90 : 5 : 5) for 3 h. The resin was removed by filtration, and the crude peptides were recovered by precipitation with cold anhydrous ethyl ether to give a white powder and then lyophilized. The crude peptide was purified by RP-HPLC, and molecular weights were determined by ESI mass spectrometry.

### 2.4. Western Blot, Immunoprecipitation, and Angiogenesis *In Vitro*

Cells were treated with 40 ng/ml of RH10 or CTRL−. Western blot, immunoprecipitation, and angiogenesis *in vitro* were all performed as previously described [26, 29–31]. *β*-Actin (C-4) (sc-47778) and I*κ*B antibodies were from Santa Cruz Biotechnology, Inc.; Myc (#2272), p-NF*κ*B (#3033), and cleaved caspase 3 (#9661) were from Cell Signaling Technology. For angiogenesis *in vitro*, endothelial cells were directly treated with RH10 or CTRL−. For tumor angiogenesis, KAT-4 cells were treated with these peptides, and the cultured medium was collected after 24 hours from starting treatment. This medium was then added to endothelial cells, and tubular formations were evaluated after 12 hours on Matrigel matrix.

### 2.5. Radiation and Chemotherapy

Cells were pretreated with 20 ng/ml of RH10 and CTRL− and then treated for 24 hours with a single dose of 4 gray (4 Gy) X-radiation at room temperature (250 KV, 16 Ma, dose rate: 1.5 Gy/min), 1 nM cisplatin, or 100 nM doxorubicin.

### 2.6. Proliferation Assay

Proliferation was evaluated using CyQUANT® NF Cell Proliferation Assay Kit (Invitrogen), following the manufacturing instructions.

### 2.7. TUNEL Assay

To evaluate apoptosis, cancer cells were first treated with RH10 and CTRL−. Then, we performed a TUNEL assay using the “DeadEnd Colorimetric TUNEL System” from Promega, following the manufacturing instructions. Images were acquired at Eclipse E1000 Fluorescence Microscope (Nikon, Milan, Italy) using SigmaScan Pro software (Jandel). Results are expressed as mean ± SD of apoptotic nuclei.

### 2.8. ROS Production

The production of reactive oxygen species (ROS) was determined through the oxidation of a cell-permeable nonfluorescent probe, 2′,7′-dichlorofluorescin diacetate (H_2_DCFDA: Sigma-Aldrich), to the fluorescent DCF as described before [32]. Briefly, KAT-4 cells were plated at a density of 5 × 10^4^ for each well in 24-well plates. After incubation with the peptide RH10 or the negative control for 24 h, the cells were incubated with 5uMH_2_DCFDA for 30 min at 37°C in a humidified atmosphere (5% CO_2_, 95% air). After the incubation, cells were washed twice with PBS and fresh medium was added. The fluorescence was immediately measured by a plate reader (Tecan Infinite200Pro) using excitation/emission wavelengths of 492/520 nm.

### 2.9. *In Vivo* Study

Experiments were carried out, in accordance to NIH guidelines for Animal Investigation, in 8-week-old BALB/c immunoincompetent nude mice (Charles River), which had access to food and water ad libitum. All *in vivo* experimental protocols were approved by the Federico II University Ethical Committee for Animal Studies. Sample size calculation showed that 5 mice/group were needed to achieve the statistical power of 0.8 based on previous *in vivo* experiments using the full-length RH sequence [26].

For tumor formation, a suspension containing 2 × 10^6^ KAT-4 cells in 200 *μ*l of DMEM was injected subcutaneously in the dorsal side of nude mice. Animals were anesthetized using isoflurane 2%. We used mice that developed a tumor with a diameter ≥ 6 mm within 2 weeks. Mice were divided into four groups (5 mice/group) and treated with intratumor injections of the specific peptide (RH10 and CTRL−) twice a week for 3 weeks. In particular, two groups received RH10 at either low (0.1 mg/kg) or high dosage (3 mg/kg), one group received the higher dose of CTRL− (3 mg/kg), and another group was treated with DMEM alone. Tumor growth was measured by a caliper twice a week. After three weeks, tumors were collected for Western blot analysis.

### 2.10. Statistical Analysis

All values are presented as mean ± SD. One-way ANOVA with a Bonferroni post hoc test was performed to compare the different parameters among the different groups. A significance level of *p* < 0.05 was assumed for all statistical evaluations. Statistics were computed with GraphPad Prism Software (San Diego, California).

## 3. Results

### 3.1. Identification of the Minimum Effective Sequence of the RH Domain

**Figure 1:**
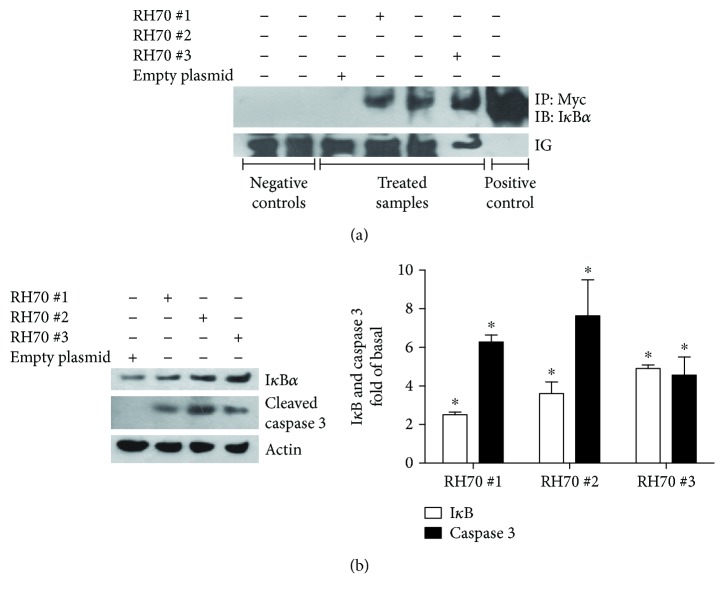
Cloning and testing of RH truncated mutants. (a) To test the ability of truncated mutants to bind I*κ*B, we performed a coimmunoprecipitation assay, using Myc tag for immunoprecipitation. The first two lines are negative controls: (1) agarose beads + Ab anti-Myc; (2) agarose beads + secondary Ab + cell lysate; lines 3–6 include agarose beads + Ab anti-Myc + lysate of cells transfected as described in the legend. Line 7 is a positive control (cell lysate). Images show the ability of the mutants to precipitate I*κ*B. Since the mutants are too small to be identified by electrophoresis, immunoglobulins (Ig) were used as a control of immunoprecipitation. The images are the representative of the results from the three independent experiments. (b) I*κ*B and caspase 3 levels were assessed by Western blot in response to mutants expression. Actin was used as loading control. All the mutants are able to increase both I*κ*B and caspase 3 levels. The images are the representative of the results from the three independent experiments. Densitometric analysis (bar graph) shows fold of increase of I*κ*B and cleaved caspase 3 levels vs. control (^∗^*p* < 0.05 vs. control). Data are reported as mean ± SD.

To identify the minimum effective sequence of RH that was able to exert its anticancer effect, we cloned three overlapping mutants of the RH and evaluated their effectiveness to regulate NF*κ*B signaling. Given the effectiveness of the full-length RH protein in KAT-4 cells [26], we performed the experiments in this cell line.  Figure 1 shows that all mutants immunoprecipitated I*κ*B (Figure 1(a)) and increased its expression (Figure 1(b)) compared with controls. Moreover, all the mutants were able to regulate NF*κ*B-dependent apoptotic events as demonstrated by increased levels of cleaved caspase 3 (Figure 1(b)).

The analysis of mutants sequence showed that they share a ten amino acids long sequence that could be responsible for RH effects on NF*κ*B signaling (Table 1). We then synthesized two peptides: one reproducing these ten identified amino acids (RH10) and another one reproducing the ten preceding ones (CTRL−). Both peptides were conjugated to the sequence of the transactivating transcriptional activator (TAT) from human immunodeficiency virus to allow the autonomous internalization of the peptides (Table 1).

### 3.2. Effect of RH10 on Tumor Cell Proliferation and Signaling

**Figure 2:**
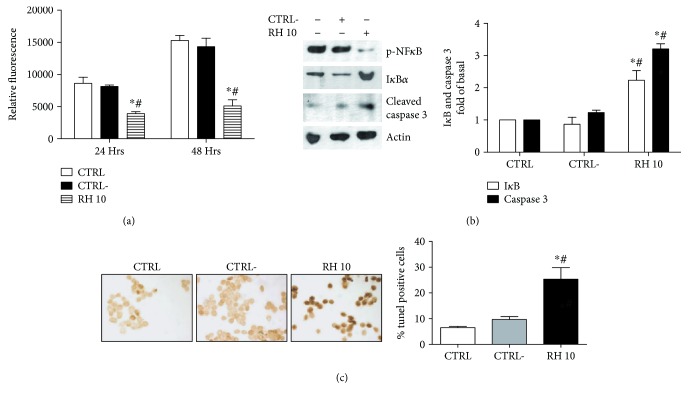
RH10 inhibits cancer cell proliferation by the modulation of NF*κ*B activity. (a) KAT-4 were treated with RH10 or CTRL−. A proliferation assay was performed 24 and 48 hours after treatment. RH10 significantly inhibits cell proliferation at both 24 and 48 hours after treatment (^∗^*p* < 0.05 vs. CTRL, #*p* < 0.05 vs. CTRL−). Data are reported as mean ± SD. (b) The effects of RH10 on NF*κ*B signaling and apoptosis were evaluated by Western blot using specific antibodies. RH10 reduces NF*κ*B activation and increases I*κ*B and caspase 3 levels. The inset shows a representative blot from three independent experiments. Data are reported as mean ± SD (^∗^*p* < 0.05 vs. CTRL; #*p* < 0.05 vs. CTRL−). (c) Apoptosis was evaluated by TUNEL assay. Cells were treated with RH10 or CTRL−. The assay was performed according to the manufacturers' instructions. The images are representative of the results from three independent experiments. Data are reported as mean ± SD (^∗^*p* < 0.05 vs. CTRL; #*p* < 0.05 vs. CTRL−).

We first evaluated the effects of RH10 and CTRL− on KAT-4 proliferation after 24 and 48 hours from treatment (Figure 2(a)). RH10 significantly reduced cell proliferation in a time-dependent manner compared with both control and CTRL−. We then evaluated the effects of the peptide on NF*κ*B signaling. RH10 was able to reduce the phosphorylation and activation of NF*κ*B and to increase I*κ*B*α* levels (Figure 2(b)). Moreover, the treatment with RH10 triggered apoptotic events as demonstrated by the increased levels of cleaved caspase 3 (Figure 2(c)) and by the TUNEL assay (Figure 2(c)). On the contrary, CTRL− had no effects on NF*κ*B signaling and its associated phenotypes, suggesting the effectiveness and specificity of the RH10 peptide.

### 3.3. Effect of RH10 on ROS Production

**Figure 3:**
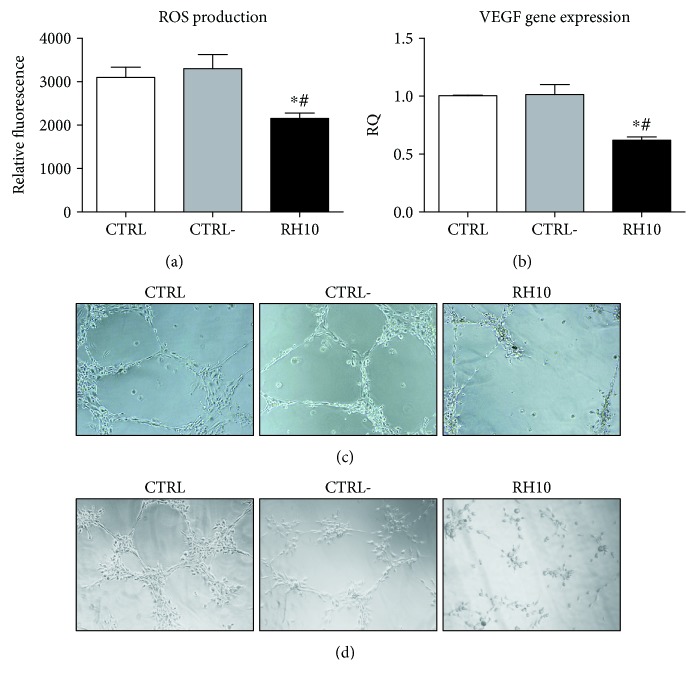
RH10 affects ROS production and angiogenesis *in vitro*. (a) KAT-4 cells were plated at a density of 5 × 10^4^ for each well in 24-well plates. After incubation with the peptide RH10 or the negative control for 24 h, the cells were incubated with 5uMH_2_DCFDA for 30 minutes, and fluorescence was immediately measured at a plate reader. RH10 was able to reduce ROS production compared with controls. Data are reported as mean ± SD (^∗^*p* < 0.05 vs. CTRL; #*p* < 0.05 vs. CTRL−). (b) VEGF gene expression was evaluated by real-time PCR. RH10 reduced VEGF expression compared with controls. Data are reported as mean ± SD (^∗^*p* < 0.05 vs. CTRL; #*p* < 0.05 vs. CTRL−). (c–d) Endothelial cells were plated on Matrigel matrix, and tubular formation was evaluated 24 hours after treatment with CTRL− or RH10. RH10 inhibits angiogenesis *in vitro* compared with control and CTRL−(c). In another set of experiments, endothelial cells were treated with supernatants from KAT-4 cells treated with peptides (d). RH10 was able to inhibit tumor angiogenesis. The images are the representative of the results from the three independent experiments.

Reactive oxygen species (ROS) levels are increased over physiological levels in cancer and are responsible for the oxidative stress that regulates tumor progression [33]. Moreover, ROS production regulates and is regulated by NF*κ*B [34]. Thus, we evaluated the effectiveness of RH10 in regulating oxidative stress in cancer cells. The treatment with RH10 inhibited cellular ROS production in KAT-4 compared with both controls (Figure 3(a)). Mitochondrial ROS levels were unaffected by the treatment with RH10 (data not shown), thus suggesting that cytosolic ROS are the ones involved in RH10-dependent signaling.

### 3.4. Effect of RH10 on Angiogenesis

Angiogenesis is one of the NF*κ*B-associated phenotypes that are responsible for tumor progression and metastatization. To confirm the effectiveness of RH10 to inhibit NF*κ*B signaling, we first evaluated VEGF gene expression by real-time PCR. VEGF expression was reduced in RH10-treated cells compared with controls (Figure 3(b)). We then evaluated the ability of RH10 to regulate endothelial cells network formation on a Matrigel substrate. Accordingly, RH10 inhibited angiogenesis *in vitro* compared with both control and CTRL− (Figure 3(c)). To assess the effect of RH10, specifically on tumor angiogenesis, we incubated endothelial cells with cultured medium from KAT-4 cells treated with RH10 or CTRL−, and endothelial cell network formations were evaluated.  Figure 3(d) shows that angiogenesis was reduced in cells incubated with cultured medium from KAT-4 treated with RH10 compared with controls.

### 3.5. Combined Therapies to Reduce Tumor Growth

**Figure 4:**
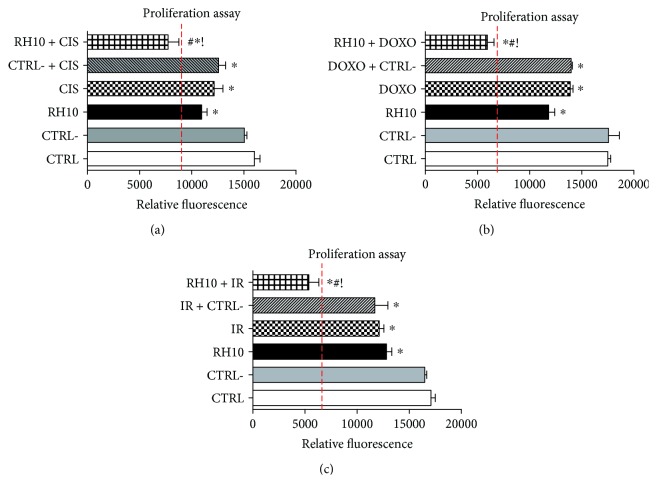
RH10 effects in combination with other therapies. KAT-4 cells were pretreated with 1 ng/ml RH10. Cell proliferation was evaluated after 24 hours of treatment with cisplatin (a), doxorubicin (b), or ionizing radiation (c). (a) RH10 further reduce cell proliferation in response to cisplatin (^∗^*p* < 0.05 vs. CTRL, #*p* < 0.05 vs. cisplatin, ! vs. RH10). Data are reported as mean ± SD. (b) RH10 further reduce cell proliferation in response to doxorubicin (^∗^*p* < 0.05 vs. CTRL, #*p* < 0.05 vs. doxorubicin, ! vs. RH10). Data are reported as mean ± SD. (c) RH10 further reduce cell proliferation in response to ionizing radiation (^∗^*p* < 0.05 vs. CTRL, #*p* < 0.05 vs. ionizing radiation, ! vs. RH10). Data are reported as mean ± SD.

To evaluate whether RH10 could be useful to sensitize cells to the treatments, we evaluated its effects on cell proliferation in combination with common used chemotherapeutic drugs (cisplatin and doxorubicin) and radiotherapy. A low dose of cisplatin (1 nM) and a lower dose of RH10 (20 ng/ml) alone were both able to reduce cell proliferation (CIS: −24 ± 4% and RH10 −31 ± 2% vs. CTRL) (Figure 4(a)). The combination of low dosages of cisplatin and RH10 further inhibited cell proliferation (RH10 + CIS −51 ± 4% vs. CTRL) (Figure 4(a)). Similarly, a low dose of doxorubicin (100 nM) reduced cell proliferation (DOXO: −20 ± 6% vs. CTRL), and the supplementation with RH10 increased such effect (RH10 + DOXO: −66 ± 7% vs. CTRL) (Figure 4(b)). We finally evaluated the effects of RH10 in response to ionizing radiation.  Figure 4(c) shows that RH10 further reduced cell proliferation in response to ionizing radiation (RH10 + IR: −68 ± 12% vs. CTRL) compared with ionizing radiation alone (IR: −29 ± 5% vs. CTRL). These results suggest that RH10 is able to sensitize cells to better respond to common cancer therapies.

### 3.6. RH10 Inhibits Tumor Growth *In Vivo*

**Figure 5:**
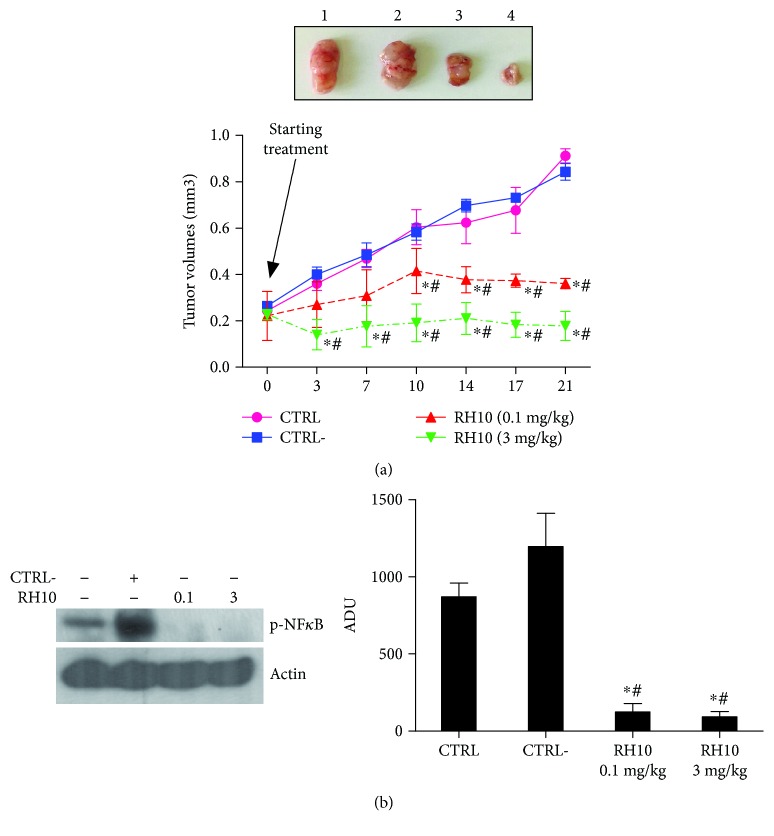
RH10 inhibits tumor growth in a dose-dependent manner. (a) Tumor cell lines were inoculated in nude mice, and tumors were treated with 3 mg/kg CTRL−, 0.1 mg/kg RH10, and 3 mg/kg RH10 twice a week for three weeks. Results are reported in the graph as mean ± SD, and a representative image is shown (^∗^*p* < 0.05 vs. CTRL, # *p* < 0.05 vs. CTRL−; ANOVA and Bonferroni post hoc test). (b) Phospho-NF*κ*B levels were evaluated by Western blot in lysates from untreated and treated tumors. RH10 reduced p-NF*κ*B levels at all dosages compared with CTRL and CTRL−. The image is the representative of the results from the three independent experiments in different tumor samples. Data are reported as mean ± SD (^∗^*p* < 0.05 vs. CTRL, #*p* < 0.05 vs. CTRL−). ADU = arbitrary densitometric units.

To confirm *in vitro* data, we tested the effectiveness of RH10 in an animal model of cancer (Figure 5). In Balb/c nude mice, the treatment with RH10 was performed twice a week for three weeks by intratumor injections.  Figure 5(a) shows that RH10 reduced tumor growth in a dose-dependent manner compared with controls. Indeed, the low dose (0.1 mg/kg) could delay tumor progression while the high dose (3 mg/kg) completely inhibited tumor growth. On the opposite, CTRL− had no effects. Tumors were then collected at the end of the treatment, and NF*κ*B activity was evaluated by Western blot. In tumors treated with RH10, the phosphorylated and active form of NF*κ*B was significantly reduced compared with controls (Figure 5(b)). These data confirm that RH10 is able to inhibit tumor growth and progression through the inhibition of NF*κ*B signaling.

## 4. Discussion

The main finding of this study is the identification of the minimum amino acidic sequence of the RH domain of GRK5 which is needed for specific inhibition of NF*κ*B activity [25–27]. Indeed, RH10 peptide, comprising only 10 amino acids of GRK5-RH sequence, is able to reduce cancer cells proliferation *in vitro* and to inhibit tumor growth *in vivo* in a dose-dependent manner by inhibiting NF*κ*B activity. RH10 is also able to sensitize cells to therapeutic treatments, such as chemotherapy and radiotherapy. Thus, our data propose RH10 as a potential therapeutic peptide in the treatment of cancer.

Therapeutic peptides are a class of peptide-based drugs able to induce a therapeutic response through the modulation of cellular targets [2]. Compared with traditional small molecules, the use of peptides offers several advantages such as high specificity and biological activity, excellent safety and tolerability, and low costs of production [35]. Indeed, during the past decade, several peptides have reached the market for the treatment of several conditions [36] such as lung injury [37, 38], autoimmune diseases [39]and, allergic disease [40]. In this context, RH10 could join the class of cell-penetrating peptides since the 10 amino acids of RH are conjugated to TAT that allows the peptide to directly enter into the cell where it specifically binds I*κ*B*α*. A limitation in using peptide-based drugs for the treatment of diseases is the short half-life of this molecule in the blood. However, recent findings propose useful strategies to enhance the *in vivo* half-life of peptides without compromising their effectiveness [41, 42], including TAT conjugation to peptides that protects them from degradation and improves their effectiveness [41]. Our peptide fits well in this context, being conjugated to TAT sequence that could favor RH10 stability also in the blood.

The inhibition of NF*κ*B signaling is an attractive goal for cancer research since this transcription factor is strictly associated with cancer development and progression favoring tumor growth and inflammatory responses and inhibiting apoptotic events [23, 43]. NF*κ*B mediates a crosstalk between inflammation and cancer mainly through the generation of inflammatory cytokines and the induction of oxidative stress that favor tumor initiation and development [44–46]. Several inhibitors have been identified to date, which are able to inhibit NF*κ*B [47], but, since they also affect other signaling pathways, none of them have reached the clinic yet. RH10 is a specific inhibitor of NF*κ*B that does not interfere with other intracellular signaling. Indeed, RH10 affects all NF*κ*B-dependent phenotypes which are responsible for tumor progression (apoptosis, cell proliferation, angiogenesis, and oxidative stress). In fact, when endothelial cells are exposed to a conditioned medium of KAT-4 cells treated with RH10, their angiogenetic capability is impaired. Among NF*κ*B-dependent phenotypes, oxidative stress via ROS production is one of the main causes of tumor progression [34]. Similarly, cancer cells are usually characterized by high levels of ROS which affect several phenotypes such as proliferation, death evasion, angiogenesis, and metastasis. Here, we show that the treatment with RH10 is able to reduce oxidative stress in cancer cells. Chemotherapy and radiations are the common strategies that are engaged to reduce tumor mass in humans. The chemotherapy comprises drugs that at specific dosages are effective to reduce tumor mass but often trigger cardio-toxic effects [48]. For this reason, new effective and nontoxic drugs are needed to be used as adjuvants in the common therapeutic regimens to sensitize cancer cells and lower the dosages of common toxic drugs. RH10 seems to be a useful adjuvant in common therapies since it primes cells to better respond to some chemotherapeutic drugs or ionizing radiation. This latter property of RH10 could be very useful for therapeutic purposes considering the multiple acute and chronic side effects that derive from chemotherapy and radiotherapy [49, 50].

It has been shown that GRK5 is involved in tumor growth and progression even if its effect is quite controversial. Indeed, GRK5 seems to have a double effect on cancer being an inhibitor or inducer of cancer progression depending on its subcellular localization and cancer type [28]. It has to be considered that GRK5 is a large multidomain kinase proven to be able to interact with many proteins and interfere with several signal transduction pathways [51]. Conversely, our peptide RH10 is only a fragment of GRK5 but retains the anticancer effects based on I*κ*B interaction and stabilization.

## 5. Conclusion

In the last decade, cancer targets have been proposed for personalized therapies. The research has a key role in the fight against cancer with the aim to identify novel no toxic drugs that are effective also in advanced stages of the disease and to identify novel biomarkers for prevention and rapid intervention. In this context, our peptide fits well since it is able to inhibit cancer cells progression through its effects on NF*κ*B signaling and its associated phenotypes (cell proliferation, apoptosis, angiogenesis, and oxidative stress). Thus, RH10 could be considered a potential specific and effective drug to be tested in clinical trials.

## Figures and Tables

**Table 1:** The amino acid sequence of RH10 and CTRL− including the TAT sequence.

Peptide name	TAT sequence	Target sequence	Full sequence
RH10	GRKKRRQRRRPQ	TPKSPVFITQ	GRKKRRQRRRPQTPKSPVFITQ
CTRL−	GRKKRRQRRRPQ	KGKEIMTKYL	GRKKRRQRRRPQKGKEIMTKYL

## Data Availability

Data used to support the findings of this study are included within the article.
